# In situ measurements of oxidation–reduction potential and hydrogen peroxide concentration as tools for revealing LPMO inactivation during enzymatic saccharification of cellulose

**DOI:** 10.1186/s13068-021-01894-1

**Published:** 2021-02-18

**Authors:** Adnan Kadić, Anikó Várnai, Vincent G. H. Eijsink, Svein Jarle Horn, Gunnar Lidén

**Affiliations:** 1 Department of Chemical Engineering, Lund University, P.O. Box 118, 221 00 Lund, Sweden; 2 Faculty of Chemistry, Biotechnology and Food Sciences, Norwegian University of Life Sciences (NMBU), P.O. Box 5003, NO-1432 Ås, Norway

**Keywords:** Oxidation reduction potential, Avicel, Hydrogen peroxide electrode, Saccharification, AA9

## Abstract

**Background:**

Biochemical conversion of lignocellulosic biomass to simple sugars at commercial scale is hampered by the high cost of saccharifying enzymes. Lytic polysaccharide monooxygenases (LPMOs) may hold the key to overcome economic barriers. Recent studies have shown that controlled activation of LPMOs by a continuous H_2_O_2_ supply can boost saccharification yields, while overdosing H_2_O_2_ may lead to enzyme inactivation and reduce overall sugar yields. While following LPMO action by ex situ analysis of LPMO products confirms enzyme inactivation, currently no preventive measures are available to intervene before complete inactivation.

**Results:**

Here, we carried out enzymatic saccharification of the model cellulose Avicel with an LPMO-containing enzyme preparation (Cellic CTec3) and H_2_O_2_ feed at 1 L bioreactor scale and followed the oxidation–reduction potential and H_2_O_2_ concentration in situ with corresponding electrode probes. The rate of oxidation of the reductant as well as the estimation of the amount of H_2_O_2_ consumed by LPMOs indicate that, in addition to oxidative depolymerization of cellulose, LPMOs consume H_2_O_2_ in a futile non-catalytic cycle, and that inactivation of LPMOs happens gradually and starts long before the accumulation of LPMO-generated oxidative products comes to a halt.

**Conclusion:**

Our results indicate that, in this model system, the collapse of the LPMO-catalyzed reaction may be predicted by the rate of oxidation of the reductant, the accumulation of H_2_O_2_ in the reactor or, indirectly, by a clear increase in the oxidation–reduction potential. Being able to monitor the state of the LPMO activity in situ may help maximizing the benefit of LPMO action during saccharification. Overcoming enzyme inactivation could allow improving overall saccharification yields beyond the state of the art while lowering LPMO and, potentially, cellulase loads, both of which would have beneficial consequences on process economics.

## Background

During the past decade, the introduction of lytic polysaccharide monooxygenases, LPMOs, in cellulase mixtures led to a marked improvement in the efficiency of saccharification of pretreated biomass [1–3]. Through their oxidative mechanism of attacking the cellulose [4–8], these enzymes provide a different mode of bond breakage than the hydrolases, i.e., the exo-acting cellobiohydrolases (CBH) and the endo-acting endoglucanases (EG). The mode of interaction between LPMOs and hydrolytic enzymes, including CBHs and EGs, has been studied by several groups (e.g., [9–12]), as reviewed recently by Østby et al. [13]. On the one hand, LPMOs, similarly to EGs, generate free chain ends upon endolytic cleavage of cellulose where CBHs can start their action and cleave off cellobiose units in a processive manner. A main difference between LPMOs and EGs is that EGs act on amorphous regions, whereas LPMOs can cleave crystalline cellulose [9]. On the other hand, oxidation at the generated reducing or non-reducing chain ends by LPMOs may positively or negatively affect substrate binding by CBHs. In addition to enzyme-specific synergistic effects, the synergism between LPMOs and cellulases may be substrate dependent, as shown by Tokin et al. [12]. From the point of biomass processing, the improved biomass accessibility by inclusion of LPMOs may come at a price of a somewhat decreased sugar yield as the action of LPMOs results in the formation of oxidized sugars (d-gluconic acid and/or 4-keto-d-glucose) upon oxidation of the terminal sugar at the newly generated chain ends. As an example, a study by Cannella et al. shows that up to 4% of the glucose solubilized from pretreated wheat straw by Cellic CTec2 ended up as d-gluconic acid [14]. Considering the importance of LPMOs for process efficiency and the possible “loss” of glucose due to oxidation, the fraction and action of LPMOs in cellulase mixtures need to be properly controlled to obtain optimal process performance. Another reason to do so relates to side reactions catalyzed by the LPMO that may damage the enzymes, as outlined below.

The oxidative action of LPMOs requires the presence of either O_2_ and stoichiometric amounts of reducing agent or lignin [4, 5] or H_2_O_2_ and priming amounts of reducing agent [6, 15, 16]. While the action of LPMOs, as shown by the formation of oxidized sugars and glucose by LPMO-containing cellulase cocktails, can be harnessed in bioreactors using both the O_2_-dependent [3, 14] and the H_2_O_2_-dependent [17] reaction mechanisms, the H_2_O_2_-dependent reaction mechanism has been shown to be more effective, both at laboratory scale [17–19] and recently also at demonstration scale [20] for lignin-poor biomass. The use of added H_2_O_2_ enables LPMO activity without the need for gas–liquid mass transfer of oxygen. This is potentially beneficial from a process perspective, since controlling the oxygen transfer rate will be more difficult than controlling the H_2_O_2_ addition. It should be noted that under aerobic conditions, H_2_O_2_ will be formed in situ from the reaction of O_2_ and externally supplied reductant [21, 22], and that O_2_ and H_2_O_2_ will be formed and consumed also through abiotic reactions with lignin [22, 23]. In order to understand the redox processes taking place in the reactor and to have control over LPMO reactions, abiotic redox reactions would need to be monitored closely during saccharification.

While in case of a lignin-poor feedstock, addition of H_2_O_2_ promotes enzymatic cellulose degradation by LPMOs, high levels of H_2_O_2_ can lead to oxidative damage of the LPMO active site and, consequently, may completely and irreversibly destroy the catalytic activity [6, 24]. For this reason, H_2_O_2_ cannot be added as a single pulse, but has to be gradually added during the process, in such a way that the dissolved H_2_O_2_ concentration in the reaction medium remains very low at all times [17]. There are indications in the literature that suitable addition rates will likely depend on factors such as the redox stability and catalytic performance of the LPMO, the substrate concentration, the extent of substrate binding and the availability of reductant [6, 25, 26]. It will, therefore, be difficult to set the rate of H_2_O_2_ feed a priori, but with some experimental work, acceptable feed profiles can be empirically determined (as exemplified in [17]). However, variations in raw material properties, enzyme activity or process conditions make this approach suboptimal and in the worst case even susceptible to failure. Methods for online monitoring of LPMO activity to safeguard the process from failure due to overfeeding of hydrogen peroxide are therefore desirable.

We have previously examined the suitability of an oxidation–reduction potential (ORP) redox electrode, which provides a “summarizing” signal about the redox state of the reaction, to monitor changes in LPMO activity during batch enzymatic saccharification of sulfite pretreated softwood pulp [27]. We found that under well-controlled laboratory-scale saccharification experiments with H_2_O_2_ feeding, there was an increase in the ORP signal at the time point when the accumulation of LPMO products came to a halt. The ORP is affected by several factors, but the observed changes in the ORP could correlate with the accumulation of dissolved H_2_O_2_ in the system, caused by decreased activity of LPMOs.

In the current study, we studied the correlation between observed changes in the ORP, dissolved H_2_O_2_ levels and the onset of LPMO inactivation. We carried out enzymatic saccharification of cellulose (Avicel) with the commercial cellulase preparation Cellic CTec3 under anaerobic conditions with different H_2_O_2_ feed rates using ascorbic acid as reductant. The actual dissolved H_2_O_2_ level was monitored using a small laboratory-scale H_2_O_2_ electrode, and also the ORP was measured using an electrode. These continuous signals were analyzed in relation to offline determined glucan conversion yields and oxidized sugar levels, which serve as good indicators of LPMO activity. Furthermore, we followed the oxidation of the reductant (ascorbic acid) spectrophotometrically in order to be able to estimate hydrogen peroxide consumed in this reaction. The concomitant monitoring of these process parameters provided quantitative insight into LPMO action and the fate of hydrogen peroxide during the cellulose degradation reaction.

## Results and discussion

### Glucan conversion

**Fig. 1:**
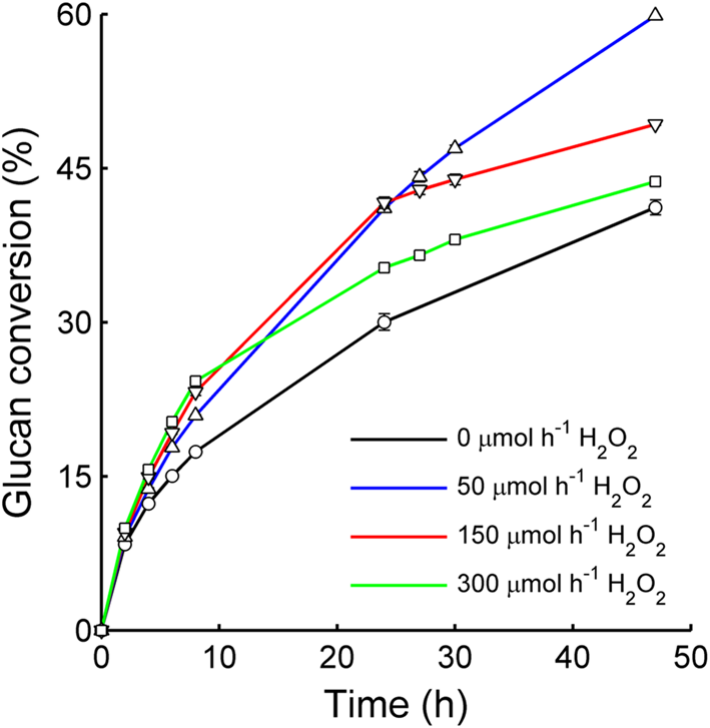
Glucan to glucose conversion during enzymatic saccharification of Avicel in anaerobic conditions with H_2_O_2_ feeding. Reactions were initially supplied with 1000 µM ascorbic acid. Feed rates used: black, 0 µmol h^−1^ H_2_O_2_; blue, 50 µmol h^−1^ H_2_O_2_; red, 150 µmol h^−1^ H_2_O_2_; green, 300 µmol h^−1^ H_2_O_2_. Standard deviations calculated from duplicate experiments are indicated by bars, but in most cases the deviation was so small that this is not visible in the figure. An additional set of experiments was also made with a feeding rate of 100 µmol h^−1^ H_2_O_2_, but glucan conversion was not measured in those experiments

All glucan degradation experiments were conducted under anaerobic conditions to avoid confounding effects from dissolved oxygen on oxidation-reduction potential measurements. Enzymatic saccharification of Avicel (at 5%, w/w) with Cellic CTec3 was performed under anaerobic conditions either without H_2_O_2_ feeding or using a feed rate of 50, 100, 150 or 300 µmol h^−1^. A pronounced effect of the H_2_O_2_ feeding was seen (Fig. 1), in agreement with previous studies [17]. The saccharification yield without H_2_O_2_ feed (0 µmol h^−1^; black curve in Fig. 1) was significantly lower than the yields in all the experiments with H_2_O_2_ feed. When looking at the initial phase of the saccharification (up to 8 h), increasing the H_2_O_2_ feed from 50 to 300 µmol h^−1^ increased the rate of saccharification. However, the saccharification rate rapidly decreased after the initial 8-h period at the higher H_2_O_2_ feed rates (150 and 300 µmol h^−1^, red and green curves in Fig. 1, respectively). After 48 h, the highest glucan conversion level (60%) was reached with the lowest H_2_O_2_ feed rate (50 µmol h^−1^). This yield is similar to what has been observed previously for Avicel saccharification at 10% (w/w) substrate loading, using similar (4 mg/g DM) Cellic CTec2 loading and a H_2_O_2_ feed rate of 90 µmol h^−1^ [17].

### Oxidation–reduction potential and H_2_O_2_ concentration

**Fig. 2:**
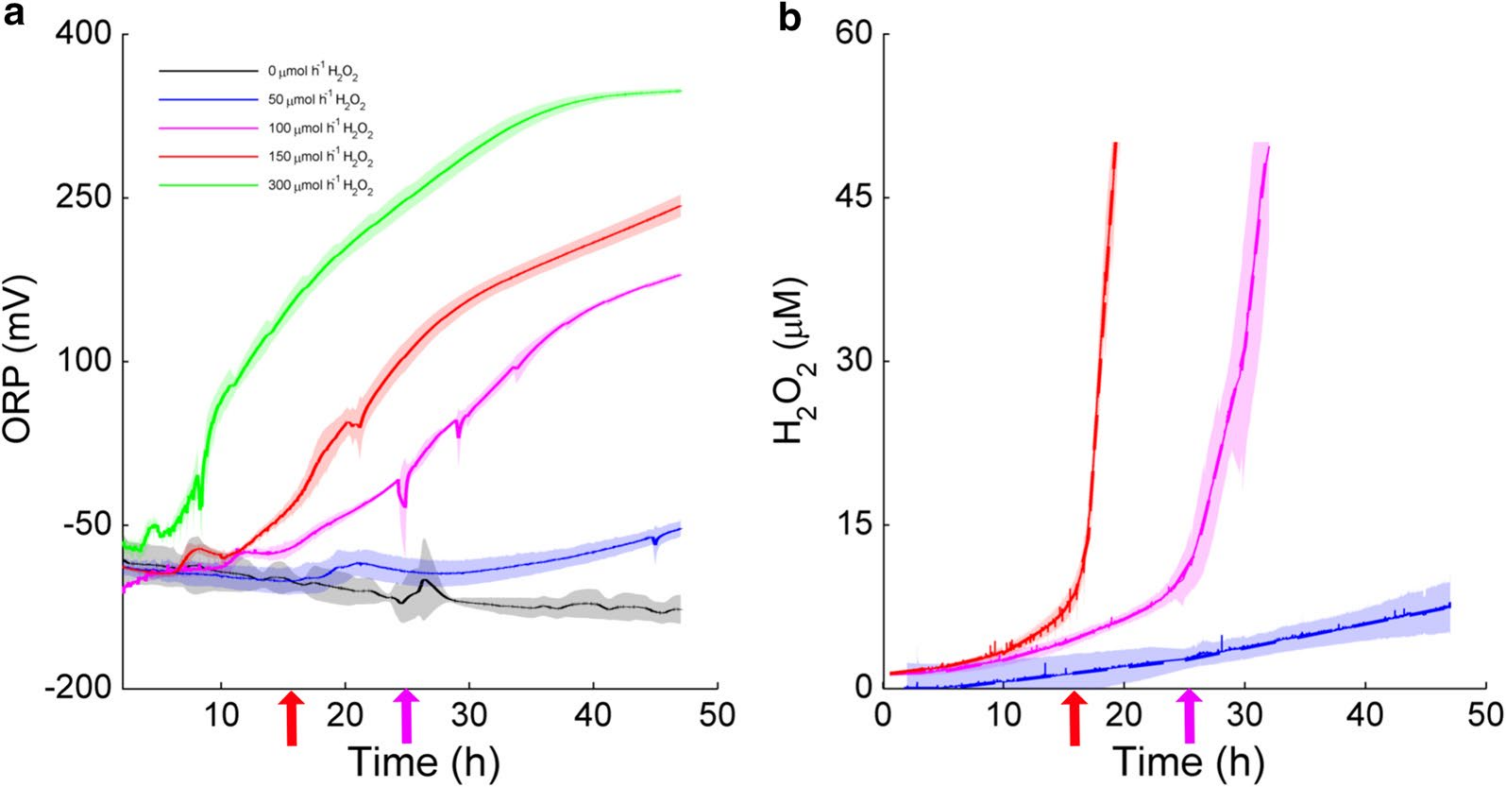
Oxidation–reduction potential (**a**) and H_2_O_2_ concentration (**b**) measured in situ during enzymatic saccharification of Avicel. Reactions were initially supplied with 1000 µM ascorbic acid and were run in anaerobic conditions with H_2_O_2_ feeding. Feed rates used: black, 0 µmol h^−1^ H_2_O_2_; blue, 50 µmol h^−1^ H_2_O_2_; magenta, 100 µmol h^−1^ H_2_O_2_; red, 150 µmol h^−1^ H_2_O_2_; green, 300 µmol h^−1^ H_2_O_2_. The shaded regions represent the standard deviation. The 300 µmol h^−1^ H_2_O_2_ feed resulted in a too rapid rise in H_2_O_2_ concentration for the electrode to be able to record the data. Accumulation of H_2_O_2_ could be followed up to 50 µM, which is the upper detection limit of the H_2_O_2_ electrode. The magenta and red arrows indicate the approximate time points where the rate of H_2_O_2_ accumulation in the medium started increasing for the reactions with 100 and 150 µmol h^−1^, respectively

Oxidation–reduction potential (ORP) and H_2_O_2_ concentration were measured in situ during enzymatic saccharification as a possible indicator of changes in LPMO activity. Without H_2_O_2_ feeding (and in the absence of oxygen), the ORP signal slightly decreased over time before stabilizing around 100 mV (Fig. 2a). The signal in this case agrees qualitatively well with measurement made in a previous work [27] on enzymatic saccharification of sulfite pretreated spruce wood, in which the naturally present SSL liquid served as a reductant, but the absolute value was higher in the present study due to a different medium background. Feeding of H_2_O_2_ increased the ORP, and higher feed rates led to an earlier and more rapid increase in the ORP (Fig. 2a).

Figure 2b shows the accumulation of H_2_O_2_ during the reactions. Without H_2_O_2_ feeding, the H_2_O_2_ concentration remained below the detection limit (not shown), whereas feeding of H_2_O_2_ led to an accumulation of H_2_O_2_. The accumulation was rather constant for the 50 µmol h^−1^ feed rate. However, for feeding at 100 and 150 µmol h^−1^, an initial slow accumulation of H_2_O_2_ accelerated substantially around 25 and 16 h, respectively. The acceleration in H_2_O_2_ accumulation occurred at the same time as the slow-down of glucan conversion in the case of the 150 µmol h^−1^ feed rate (compare Figs. 1 and 2b). The slow-down of glucan conversion is likely due to autocatalytic inactivation of the LPMOs, which is expected to occur under these conditions [6, 17], and may also relate to a more general increase in oxidative damage of other enzymes in the cellulase cocktail [28], for example due to the formation of hydroxyl radicals through reactions of H_2_O_2_ with transition metals in the solution.

The ORP signal starts increasing before the rapid H_2_O_2_ accumulation takes place (see curves for 100 and 150 µmol h^−1^ H_2_O_2_ in Fig. 2), indicating that the ORP could work as an early warning signal for H_2_O_2_ accumulation and potential LPMO inactivation as suggested by Kadić et al. [27]. The ORP is typically a logarithmic function of concentration, and regression of the measurement data gave the correlation:

$$ {\text{ORP}} = - 120.2 + 39.14 {\text{ln}}\left[ {{\text{H}}_{2} {\text{O}}_{2} } \right]. $$

**Fig. 3:**
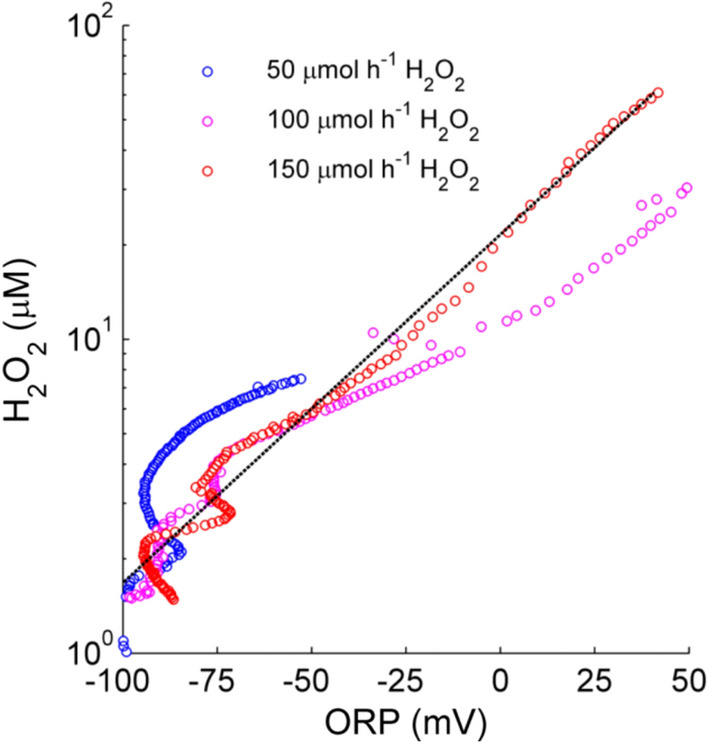
Correlation between the oxidation–reduction potential (ORP) and H_2_O_2_ concentration during enzymatic saccharification of Avicel. Reactions were initially supplied with 1000 µM ascorbic acid and were run in anaerobic conditions with H_2_O_2_ feeding. Feed rates used: blue, 50 µmol h^−1^ H_2_O_2_; magenta, 100 µmol h^−1^ H_2_O_2_; red, 150 µmol h^−1^ H_2_O_2_. The black line represents the fitted function: $\mathrm{ORP}=-120.2+39.14$ In [H_2_O_2_] with *R*^2^ = 0.87

The quality of the fit (*R*^2^ = 0.87) indicates that, at least for a simple model system of enzymatic saccharification under anaerobic conditions, the ORP can be used to estimate the H_2_O_2_ concentration in the reactor. Deviations were higher at low H_2_O_2_ concentrations (Fig. 3), likely because the relative contribution of other redox-active compounds to the ORP was larger. Using the ORP electrode is advantageous since this electrode is much more robust than the H_2_O_2_ electrode and can be used for more complex media and at higher substrate loadings.

### Ascorbic acid oxidation

**Fig. 4:**
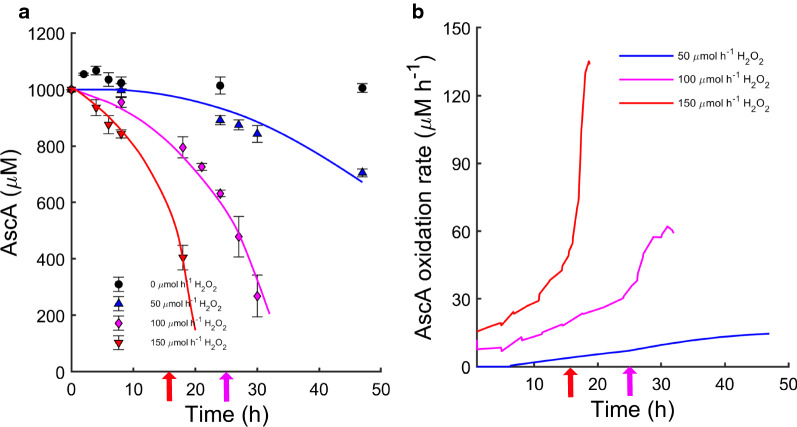
Concentration (**a**) and calculated oxidation rate (**b**) of ascorbic acid during enzymatic saccharification of Avicel. Reactions were initially supplied with 1000 µM ascorbic acid and were run under anaerobic conditions with H_2_O_2_ feeding. Feed rates used: black symbols, no feeding of H_2_O_2_; blue, 50 µmol h^−1^ H_2_O_2_: magenta, 100 µmol h^−1^ H_2_O_2_: red, 150 µmol h^−1^ H_2_O_2_. The straight lines in panel (**a**) are trendlines obtained from fitting a kinetic model assuming a first-order reaction for the rate of ascorbic acid oxidation (see Materials and methods). The trendlines were used for calculation of oxidation rates in panel (**b**), using discrete steps of a few hours. The magenta and red arrows indicate the approximate time points where the rate of H_2_O_2_ accumulation in the medium started increasing for the reactions with 100 and 150 µmol h^−1^, respectively

Ascorbic acid (AscA) is a well-known reductant, which has previously been shown to work well for priming LPMOs in H_2_O_2_-driven reactions [6] and to serve as a reductant in O_2_-driven reactions [17, 29]. In the absence of H_2_O_2_ feeding, we did not observe a decrease in the ascorbic acid concentration (Fig. 4). In contrast, the AscA concentration decreased significantly during experiments with H_2_O_2_ feeding (Fig. 4), due to oxidation of AscA by H_2_O_2_ [30]. It was not possible to determine what fraction of AscA was consumed in priming the LPMO enzymes (reduction of the catalytic copper center from Cu^2+^ to Cu^+^) and what fraction was directly oxidized by the H_2_O_2_. However, the data clearly show that higher H_2_O_2_ feed rates led to a more rapid consumption of AscA (Fig. 4), especially after the time points at which H_2_O_2_ concentrations in the medium started increasing rapidly. The increase in AscA consumption rate after these time points thus seems correlated to increasing concentrations of H_2_O_2_ in the medium (compare Figs. 2b and 4b). In the experiments with high H_2_O_2_ feed rate (100 and 150 µmol h^−1^), the highest AscA oxidation rates eventually approached the rate of H_2_O_2_ feed (see Fig. 4b). The combination of this trend in the AscA oxidation rate with observations described above suggests that when H_2_O_2_ consumption by LPMO-catalyzed cellulose oxidation had stopped, a substantial fraction of H_2_O_2_ was consumed for oxidizing AscA.

### H_2_O_2_ accumulation and cessation of LPMO action

**Fig. 5:**
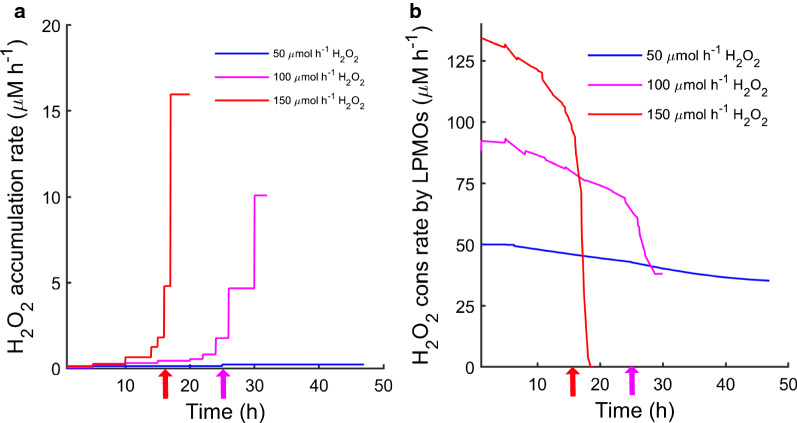
H_2_O_2_ accumulation rate (**a**) and predicted “H_2_O_2_ consumption rate by LPMOs” (**b**) during enzymatic saccharification. Reactions containing 5% (w/v) Avicel were initially supplied with 1000 µM ascorbic acid and were run under anaerobic conditions with H_2_O_2_ feeding. Feed rates used: blue, 50 µmol h^−1^ H_2_O_2_; magenta, 100 µmol h^−1^ H_2_O_2_; red, 150 µmol h^−1^ H_2_O_2_. The rate of H_2_O_2_ accumulation was determined from a piecewise linear fit of the H_2_O_2_ concentration (in Fig. 2). The predicted H_2_O_2_ consumption rate by LPMOs was calculated as the difference of H_2_O_2_ feed rate and the sum of oxidation rate of AscA and accumulation rate of H_2_O_2_. The magenta and red arrows indicate the approximate time points where the rate of H_2_O_2_ accumulation in the medium started increasing for the reactions with 100 and 150 µmol h^−1^, respectively

The two primary reactions leading to consumption of the added H_2_O_2_ are the LPMO reaction and oxidation of the reductant. If these reactions do not occur, or are too slow relative to the feeding rate, H_2_O_2_ will accumulate in the medium. The initial rate of H_2_O_2_ accumulation, calculated from the data shown in Fig. 2b, was low (Fig. 5a) but increased gradually during the experiments with H_2_O_2_ feed > 50 µmol h^−1^. Importantly, in the initial phase of the reaction, the rate of H_2_O_2_ accumulation (Fig. 5a) and the rate of ascorbic acid oxidation (Fig. 4b) were both much lower that the H_2_O_2_ feed rate. This shows that most of the added H_2_O_2_ is used by the LPMO, and that H_2_O_2_ consumption by the LPMO reaction outcompetes the reaction between H_2_O_2_ and ascorbic acid. In the experiments with H_2_O_2_ feed rates of 100 and 150 µmol h^−1^, the H_2_O_2_ accumulation rate accelerated with time (Figs. 2b and 5a), indicating that the consumption of H_2_O_2_ by the LPMOs gradually slowed down.

Despite the seemingly rapid accumulation of H_2_O_2_ close to the turning point in the reactors with 100 and 150 µmol h^−1^ H_2_O_2_ feed rates, the now relatively high accumulation rate of H_2_O_2_ was still low in comparison to its feed rate. The observed rates of 10 and 15 µM h^−1^, for the reactions with 100 and 150 µmol h^−1^, respectively, correspond to merely 10% of the H_2_O_2_ feed rates.

Next, we calculated the proportion of the H_2_O_2_ feed that neither remained unreacted in the reactor nor was consumed by oxidation of ascorbic acid (assuming a 1:1 stoichiometry in the ascorbic acid oxidation reaction). The resulting calculated rate of H_2_O_2_ conversion may be attributed to the LPMO-catalyzed reaction, and we therefore refer to it as predicted “H_2_O_2_ consumption rate by LPMOs” in Fig. 5b. Initially this rate was almost equal to the H_2_O_2_ feed rate, further supporting the notion that most of the added H_2_O_2_ was used by the LPMOs. For all H_2_O_2_ feed rates, there was a gradual decrease in the H_2_O_2_ consumption rate attributed to LPMOs, with a corresponding increase in AscA consumption (Figs. 4b and 5b). Possible causes for this gradual decrease are discussed below. In the experiments with H_2_O_2_ feed rates of 100 and 150 µmol h^−1^, the H_2_O_2_ consumption rate attributed to LPMO activity sharply fell at the turning points, which coincided with a halt in LPMO activity, as shown below.

**Fig. 6:**
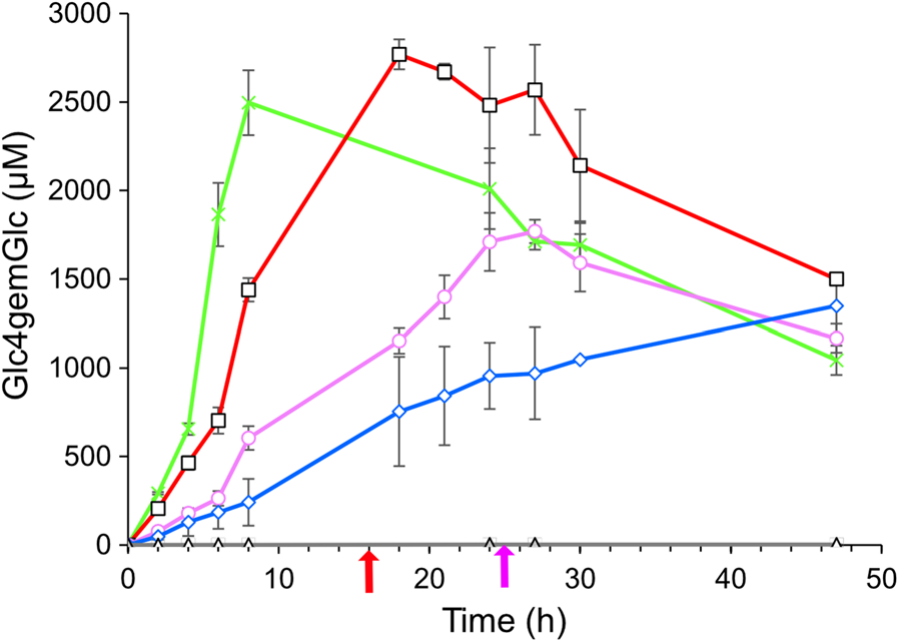
Measured C4-oxidized products (Glc4gemGlc) during saccharification of Avicel in anaerobic conditions with H_2_O_2_ feeding. Reactions were initially supplied with 1000 µM ascorbic acid. Feed rates used: grey, no added H_2_O_2_; blue, 50 µmol h^−1^ H_2_O_2_; magenta, 100 µmol h^−1^ H_2_O_2_; red, 150 µmol h^−1^ H_2_O_2_; green, 300 µmol h^−1^. The magenta and red arrows indicate the approximate time points where the rate of H_2_O_2_ accumulation in the medium started increasing for the reactions with 100 and 150 µmol h^−1^, respectively

To assess if the proportion of consumed H_2_O_2_ that was not used to oxidize ascorbic acid can be correlated quantitatively with LPMO activity, we quantified C4-oxidized sugars in the reactor setups with different H_2_O_2_ feed rates (Fig. 6). As discussed previously [17], the main detected products from LPMO activity in the Cellic products were the C4-oxidized dimer during the reactions. Quantification of this compound is prone to errors, in part due to compound instability, but it is possible to obtain reasonable estimates (see [17] for a detailed discussion).

As expected, no oxidized sugars were found in the absence of H_2_O_2_ addition. For the other reactions, there was a clear correlation between feed rates and production of oxidized sugars: the more H_2_O_2_ was added, the higher the levels of oxidized sugar were. This is similar to what has been observed before [17], where also high feed rates eventually resulted in a halt in accumulation of oxidized sugars, followed by a decline in concentration. This decline in the concentration of the C4-oxidized dimer is due to the instability of this compound, and this only becomes visible when LPMO activity ceases. These peaks in oxidized sugar levels (Fig. 6) happened at the same time as glucan solubilization became slower (Fig. 1), H_2_O_2_ started accumulating rapidly (Fig. 2b) and the oxidation of AscA rapidly increased (Fig. 4b). This loss of LPMO activity is also clearly illustrated in the calculated (predicted) “H_2_O_2_ consumption rate by LPMOs”, which drops (Fig. 5b) as the level of oxidized sugars peaks (Fig. 6).

**Fig. 7:**
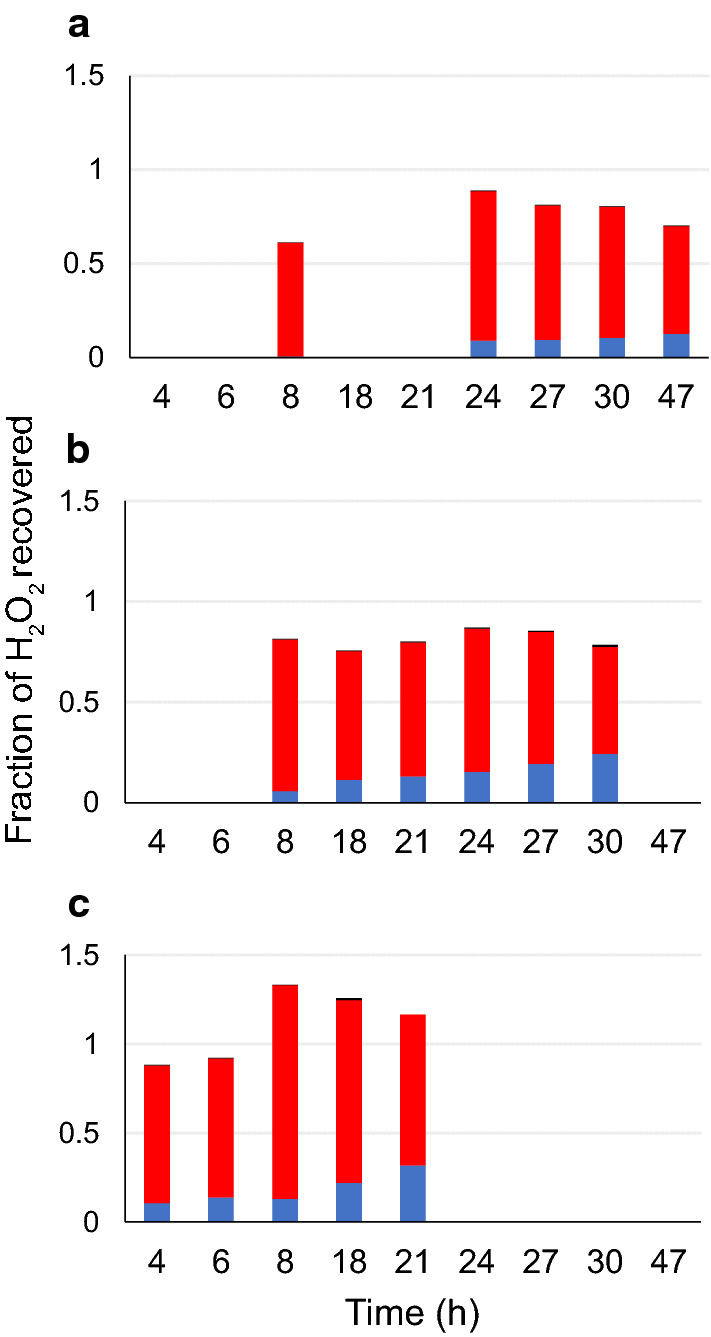
Recovered H_2_O_2_ as a fraction of totally added H_2_O_2_. The calculations were made for feed rates 50 µmol h^−1^ H_2_O_2_ (**a**), 100 µmol h^−1^ (**b**) and 150 µmol h^−1^ (**c**), until the point where the dissolved concentration of H_2_O_2_ exceeded the range of the electrode. Red bar, fraction of H_2_O_2_ consumed to generate Glc4gemGlc; blue bar, fraction of H_2_O_2_ consumed in oxidation of AscA; black bar, fraction of H_2_O_2_ remaining in liquid (this is at most about 1% and thus barely visible in the graph)

Given the precision of the measurements, the recovery of total added H_2_O_2_ in terms of generated oxidized sugars, oxidized ascorbic acid and remaining H_2_O_2_ in the medium (Fig. 7) closed reasonably well up to the turning point for the reactions with 100 and 150 µmol h^−1^ feed, although the recovery was a bit high for the latter case. For the lowest feed rate, 50 µmol h^−1^, the levels of oxidized products were lower than expected in the later phase of the saccharification reaction. This is likely due to the degradation of the C4-oxidized product, which is known to occur with time [17], and which is also observed for the other experiments in the phase after the LPMO activity has come to a stop (Fig. 6).

Figures 5b and 4b show a gradual decrease in H_2_O_2_ consumption rate attributed to LPMOs and a gradual increase in AscA oxidation rate long before the turning point and the start of H_2_O_2_ accumulation. Multiple, interconnected, processes could explain this observation. As the surface of the substrate is becoming oxidized, the effective substrate concentration becomes lower and LPMOs may on average spend more time in solution. In solution, reduced LPMOs are prone to re-oxidation in a futile reaction with H_2_O_2_ that generates reactive oxygen species, most likely hydroxyl radicals, that damage amino acids in the enzyme catalytic center and thus lead to enzyme inactivation [6, 16, 26]. Furthermore, re-oxidation of the LPMO would lead to consumption of AscA (which, indeed, also gradually increases; Fig. 4b), since AscA will be used in re-reduction of the LPMO. Of note, the gradual decrease in the H_2_O_2_ consumption rate attributed to LPMOs in the initial phase of the reaction (Fig. 5b) was not accompanied by a detectable decrease in LPMO activity (Fig. 6). Together, these observations may be taken to indicate that only a part of the LPMOs were needed to convert the available H_2_O_2_ to oxidized cellulose, while the other fraction of LPMOs was involved in futile cycles of oxidation and reduction, which would be accompanied by slow autocatalytic inactivation. This inactivation is likely masked by the high catalytic activity of remaining active LPMOs until the point when the amount of active LPMOs becomes limiting in the reaction. Apparent LPMO catalytic rates observed in cellulose degradation reactions are average rates and may thus reflect an average of a mixture of inactive and active LPMOs. Indeed, apparent reaction rates of LPMOs deduced from cellulose degradation reactions similar to those described here [17], were orders of magnitude lower than the maximum catalytic rates found for pure LPMOs in proper enzyme kinetics studies [18, 19, 25]. This supports the notion that in these H_2_O_2_-limited reactions, only a fraction of the LPMOs is needed to utilize the available co-substrate.

Looking at the cellulose saccharification curves of Fig. 1 and the LPMO activity curves of Fig. 6, it is clear that there is a correlation between the two. It remains to be determined whether the correlation between the cessation of LPMO activity and reduced glucan conversion is solely due to reduced LPMO activity or whether less specific H_2_O_2_-dependent oxidative damage to other members of the cellulolytic enzyme cocktail also plays a role. The latter processes will become more likely as the LPMO becomes inactivated, since this will lead to increased H_2_O_2_ levels in the solution and perhaps increased levels of (previously LPMO-bound) free copper ions. No matter these uncertainties, it seems clear that good control of the LPMO reaction is required to achieve optimal processes. An ideal reaction setup would allow in situ monitoring of a diagnostic signal that could predict LPMO inactivation before the reaction collapses (the turning point). Based on our data, warning signs could be an accelerating increase in the ORP signal (Fig. 2a), a radical decrease in the reductant concentration (Fig. 4a) or an increase of H_2_O_2_ concentration in the reaction mixture (Fig. 2b).

An interesting observation is that the turning point observed in the reactions described above appears to occur after approximately the same amount of hydrogen peroxide had been added [about 2500 µmol; the turning point was estimated from the oxidized sugar maximum in the case of 300 µmol h^−1^ (about 8 h) and the measured H_2_O_2_ concentrations as well as the oxidized sugar maxima in the case of 100 and 150 µmol h^−1^ (16 and 26 h, respectively)]. This observation may indicate that, under the present reaction conditions, the LPMOs have a limited lifespan in terms of the total number of turnovers which could also relate to the substrate being limiting. With respect to the latter, it is worth noting that higher maximum levels of oxidized sugars were obtained in previously described reactions, done under similar conditions, but with twice the amount of substrate [17]. In both cases, the maximum levels of oxidized sugars correspond to oxidation of some 1% of the glucose. Considering a possible limitation in turnover numbers, and, more generally, considering when during the reaction cellulases would benefit most from LPMO action, the question arises at what time during the saccharification reaction should the turnover capacity of the LPMOs be spent? Clearly, in this study it was beneficial to maintain LPMO activity throughout the saccharification. However, the control problem is a dynamic optimization problem, and a simple set-point control may in fact be the wrong strategy. Previous work (in aerobic conditions) has shown that it can be beneficial to start the H_2_O_2_ feed at a later stage in the saccharification and “save” the LPMO action to when it is more needed [27]. It is thus likely, that there is still a substantial potential to make even better use of the LPMOs in an enzyme cocktail by a smarter design of the hydrogen peroxide feed. Measurement of the ORP will be a useful online tool to diagnose LPMO activity.

### Conclusions

In this work, we have for the first time demonstrated by measurements in situ that the concentration of H_2_O_2_ remains below a few µM during anaerobic enzymatic saccharification of cellulose with H_2_O_2_ feeding for as long as the LPMOs are active. The oxidation–reduction potential qualitatively correlated with the measured H_2_O_2_ concentration and the decreased LPMO activity and proved its tentative usefulness as an online tool for feeding optimization in LPMO-assisted enzymatic saccharification.

While these results are promising and provide fundamental insight into the LPMO reaction, and its interplay with cellulases during cellulose conversion, further work is needed to see if and how one can monitor and optimize LPMO action in reactions with less well-defined, lignin-rich substrates. The abundance of redox-active compounds in such reactions will lead to a multitude of (possible) side reactions that likely cannot be monitored or controlled using the approaches described above [17, 22, 31]. In fact, so far, saccharification of lignin-rich substrates with controlled supply of external H_2_O_2_ has not been particularly successful [17, 32]. The present results suggest that improved harnessing of the potential of LPMOs should be possible also for this type of substrates.

## Materials and methods

### Enzymatic saccharification in a bioreactor

Saccharification experiments were carried out in a 3-L bioreactor (Biostat A Plus, Sartorius, Melsungen, Germany) with 1-L working volume containing Avicel PH-101 (Sigma Aldrich) (5%, w/v DM), ascorbic acid (1 mM) and Cellic CTec3 (0.04 g of enzyme solution/g DM Avicel) in Na-citrate buffer (50 mM at pH 5.0). Mixing was provided by a pitched-blade impeller (3 blades, 70 mm diameter) stirring at 50 rpm. The temperature was controlled at 50 °C. Anaerobic conditions were maintained by sparging the working volume before enzyme addition with 1 L/min N_2_ gas (stirring at 300 rpm) until the ORP reached a constant value. At this point the sparging was stopped and the flushing of the headspace with 1 L/min N_2_ gas was started. After enzyme addition, the flushing of the headspace was continued for the rest of the saccharification. Aqueous solutions of H_2_O_2_ were prepared from 30% hydrogen peroxide (EMSURE®, Merck, Darmstadt, Germany) at the appropriate concentrations to give a molar feed rate of 0, 50, 100, 150 or 300 µmol h^−1^, respectively, at a volumetric feed rate of 0.5 mL h^−1^. The H_2_O_2_ solution was fed with a submerged needle using a Watson-Marlow 120 peristaltic pump (Watson-Marlow, Falmouth, Cornwall, England). The feed was started 30 min after enzyme addition (*t* = 0.5 h). Duplicate experiments were made.

### In situ ORP measurement

The oxidation–reduction potential (ORP) in the bioreactor was measured with a combination redox electrode (Pt4805-DPAS-SC-K8S/200, Mettler-Toledo, Greifensee, Switzerland) consisting of a Pt working electrode and an Ag/AgCl/KCl equivalent reference electrode. The redox electrode was connected to a transmitter (Multi-parameter Transmitter M300, Mettler-Toledo, Greifensee, Switzerland) that provided the voltage reading. The ORP is shown relative to the reference electrode. The ORP relative to the standard hydrogen electrode (SHE) can be calculated according to: ORP_SHE_ = ORP + 187.6 mV.

### In situ H_2_O_2_ measurement

The H_2_O_2_ concentration in the bioreactor was measured using a sensor (ISO-HPO-2, World Precision Instruments, Sarasota, FL, USA) and a measurement unit (TBR4100/1025, World Precision Instruments, Sarasota, FL, USA). The sensor is based on single-potential amperometry, i.e., a constant potential (+ 450 mV) is applied to the working electrode. H_2_O_2_ diffusing through the gas-permeable membrane covering the electrode is oxidized on the electrode surface producing a small current that is proportional to the H_2_O_2_ concentration. Before each reaction, a 5-point linear calibration of the sensor (0, 1, 5, 10 and 50 µM H_2_O_2_) was performed in 200 mL of 50 mM Na-citrate buffer (pH 5.0) at 50 °C (See Additional File 1: Table S1; Fig. S1). Due to the working range of the instrument, the electrode was not used to follow concentrations larger than 50 µM H_2_O_2_.

### Analysis of monosaccharides

Samples of the saccharification liquid in 2-mL Eppendorf tubes were separated by centrifugation at 14,850 × *g* for 1 min at room temperature. The supernatants were filtered through 0.2-μm filters and stored at -20 °C until analysis. Concentrations of monomeric sugars were measured by HPLC (high-performance liquid chromatography). In short, the sugars were separated on a polymer column (Aminex HPX-87P, Bio-Rad Laboratories, München, Germany) at 85 °C. Deionized water was used as eluent at a flow rate of 0.6 mL min^−1^. The sugars were detected with a refractive index detector (Waters 2410, Waters, Milford, MA, USA).

### Analysis of oxidized sugars

Native and oxidized cello-oligosaccharides were analyzed using high-performance anion exchange chromatography with pulsed amperometric detection (HPAEC-PAD) on a Dionex ICS-5000 system equipped with a CarboPac PA200 analytical column (3 × 250 mm) and a CarboPac PA200 guard column (3 × 50 mm), operated with 0.1 M NaOH (A) and 0.1 M NaOH with 1 M Na-acetate (B) as eluents and a flow rate of 0.5 mL/min. LPMO products were eluted using a stepwise 26-min gradient, increasing the Na-acetate concentration from 0 to 0.055 M Na-acetate linearly in 3 min, from 0.055 to 0.15 M Na-acetate logarithmically (using type 4 convex gradient) in 6 min, and from 0.15 to 1 M Na-acetate exponentially (using type 8 concave gradient) in 11 min, then the Na-acetate concentration was decreased from 1 to 0 M Na-acetate linearly in 0.1 min and maintained at 0 M for 5.9 min to recondition the column before injecting the next sample. Chromatograms were recorded and analyzed with Chromeleon version 7.2.9 (Thermo Fisher Scientific Inc.). C4-oxidized dimers (Glc4gemGlc) were quantified as described previously by Müller et al. [3].

### Analysis of ascorbic acid

Samples of the saccharification liquid in 2-mL Eppendorf tubes were separated by centrifugation at 14,850 × *g* for 1 min. The supernatants were filtered through 0.2-μm filters and stored (< 15 min) in the dark on ice. AscA was measured spectrophotometrically at 265 nm (Helios γ, Unicam, Cambridge, UK) using a linear standard for quantification (5 to 100 µM). A solution containing 2.25 g enzyme solution per L of 50 mM Na-citrate buffer (pH 5.0) was used as a blank for the samples taken from the reactor. Trendlines for the concentrations of ascorbic acid (Fig. 4a) were obtained from a fitted kinetic expression assuming a first-order reaction for the rate of ascorbic acid oxidation with respect to both hydrogen peroxide and ascorbic acid, i.e., $- \frac{{d\left[ {{\text{Asc}}} \right]}}{dt} = k\left[ {{\text{Asc}}} \right]\left[ {{\text{H}}_{2} {\text{O}}_{2} } \right]$. Measured concentrations of ascorbic acid from samples and online hydrogen peroxide measurements were used in the parameter fitting. The rates shown in Fig. 4b were calculated from a piecewise discretization of the trendlines, using discrete steps of a few hours.

## Supplementary Information

**Additional file1**: **Table S1.** Example of calibration, showing measured currents for the two HPO electrodes used at different hydrogen peroxide concentrations. **Figure S1**. Example of sensor calibration. Plotted measured currents vs H_2_O_2_ concentrations from Table S1 and the least squares regression lines (R^2^ > 0.999) (DOCX 18 KB)

## Data Availability

The datasets used during the current study are available from the corresponding author on reasonable request.
