# Supplementary material for: In situ measurements of oxidation–reduction potential and hydrogen peroxide concentration as tools for revealing LPMO inactivation during enzymatic saccharification of cellulose
Source: Biotechnol Biofuels. 2021 Feb 18;14:46. doi: 10.1186/s13068-021-01894-1 (PMC7893893; doi:10.1186/s13068-021-01894-1)
Supplement: Supplementary file 1 — Additional file1: Table S1. Example of calibration, showing measured currents for the two HPO electrodes used at different hydrogen peroxide concentrations. Figure S1. Example of sensor calibration. Plotted measured currents vs H2O2 concentrations from Table S1 and the least squares regression lines (R2 > 0.999) (DOCX 18 KB) [file 13068_2021_1894_MOESM1_ESM.docx]

Additional file 1.

Calibration of H_2_O_2_ electrodes

Two electrodes were used for determining H_2_O_2_ concentrations in-situ during enzymatic hydrolysis. The electrodes were amperometric electrodes (ISO-HPO-2, World Precision Instruments, Sarasota, FL, USA). Calibrations were made at five different concentrations H_2_O_2_ of in the range 0-50 μM and at each concentration the current was measured. The response was highly linear within this range, and a linear model showed very high R^2^ value in all cases. However, the electrodes needed to be calibrated individually, and recalibrated before each run. Example of a calibration of the electrodes before experiment is shown in Table 1 and Fig 1.

Table S1. Example of calibration, showing measured currents for the two HPO electrodes used at different hydrogen peroxide concentrations.

| H_2_0_2_ (µM) | I (nA) sensor1 | I (nA) sensor2 |
| --- | --- | --- |
| 0 | 0.85 | 0.76 |
| 1 | 1.03 | 1.04 |
| 5 | 1.67 | 1.85 |
| 10 | 2.43 | 2.82 |
| 50 | 8.81 | 9.99 |

Figure S1. Example of sensor calibration. Plotted measured currents vs H_2_O_2_ concentrations from Table S1 and the least squares regression lines (R^2^ > 0.999).
